# Benign Hypertrophied Column of Bertin Mimicking a Renal Tumor in a Child: A Diagnostic Challenge

**DOI:** 10.7759/cureus.89434

**Published:** 2025-08-05

**Authors:** Fatima Ezzahra Oufquir, Bouchra Dahmani, Asmae Oulad Amar, Siham Alaoui Rachidi

**Affiliations:** 1 Radiology, Mohammed VI University Hospital, Tangier, MAR

**Keywords:** ceus, computed tomography, hypertrophied column of bertin, pediatric renal mass, renal pseudotumor, ultrasound

## Abstract

The hypertrophied column of Bertin (HCB) is a benign anatomical variant of the renal cortex that may mimic a neoplastic mass, particularly on ultrasound, potentially leading to unnecessary diagnostic or surgical interventions. We report the case of a nine-year-old girl in whom a renal lesion was incidentally discovered during follow-up imaging for a post-traumatic subcapsular hematoma. Renal ultrasound revealed an isoechoic mass in the mid-portion of the left kidney, extending into the renal sinus. Color Doppler demonstrated a vascular pattern similar to the adjacent parenchyma. Contrast-enhanced computed tomography (CT) confirmed the diagnosis of HCB based on key imaging features, including homogeneity, isoattenuation relative to the renal cortex, and preservation of normal renal architecture. No signs of necrosis, distortion, or abnormal enhancement were observed. This case highlights the importance of recognizing the characteristic imaging features of HCB to prevent misdiagnosis and avoid unnecessary intervention. In asymptomatic patients, conservative management with imaging follow-up is an appropriate and safe approach.

## Introduction

The column of Bertin represents a normal extension of renal cortical tissue between the renal pyramids. In some individuals, this tissue may appear enlarged and protrude into the renal sinus - a benign anatomical variant known as a hypertrophied column of Bertin (HCB). Although harmless, HCB can mimic renal neoplasms, particularly in pediatric patients, whose kidneys are smaller and more susceptible to anatomical variations [1,2]. Misinterpretation may lead to unnecessary biopsies or surgical intervention.

We present the case of a pediatric patient in whom an HCB was initially suspected to be a renal tumor on ultrasound. Cross-sectional imaging with contrast-enhanced computed tomography (CT) clarified the diagnosis and helped avoid invasive management. This case highlights the key imaging features that distinguish this common anatomical variant from true renal neoplasms.

## Case presentation

A nine-year-old girl underwent follow-up imaging for a previously diagnosed post-traumatic subcapsular hematoma of the left kidney. She was clinically asymptomatic, with no flank pain, hematuria, or signs of urinary tract infection. Physical examination was unremarkable, and laboratory investigations, including serum creatinine and estimated glomerular filtration rate (eGFR), were within normal limits.

**Figure 1:**
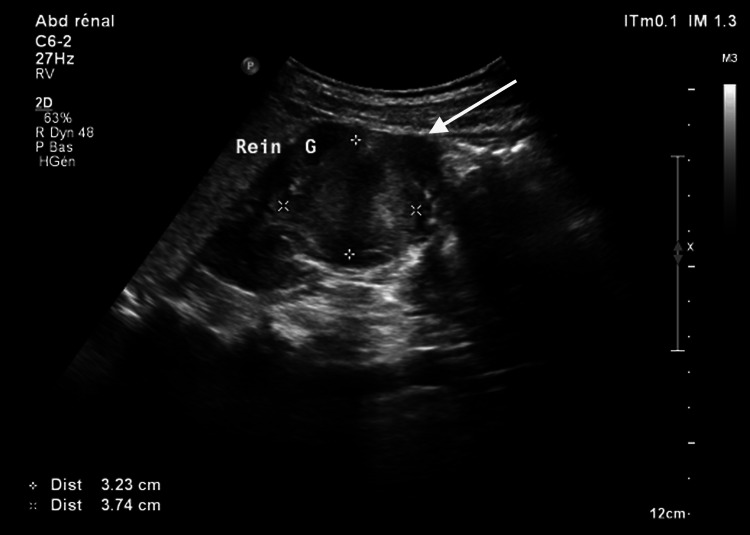
Longitudinal ultrasound image of the left kidney showing a well-defined isoechoic cortical mass extending into the renal sinus, measuring approximately 3.2 × 3.7 cm (white arrow)

Renal ultrasound revealed a well-defined, isoechoic lesion measuring 37 × 32 mm in the mid-pole of the left kidney, extending from the cortex into the renal sinus (Figure 1). Color Doppler imaging demonstrated a vascular pattern comparable to the adjacent renal parenchyma, with no abnormal internal flow.

**Figure 2:**
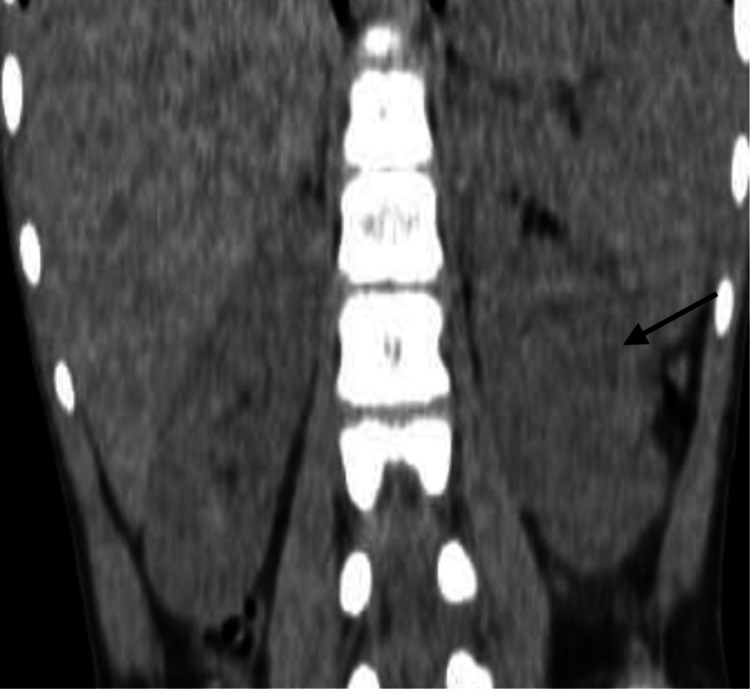
Coronal non-contrast CT of the left kidney showing a mid-polar lesion isodense to adjacent cortex (arrow)

**Figure 3:**
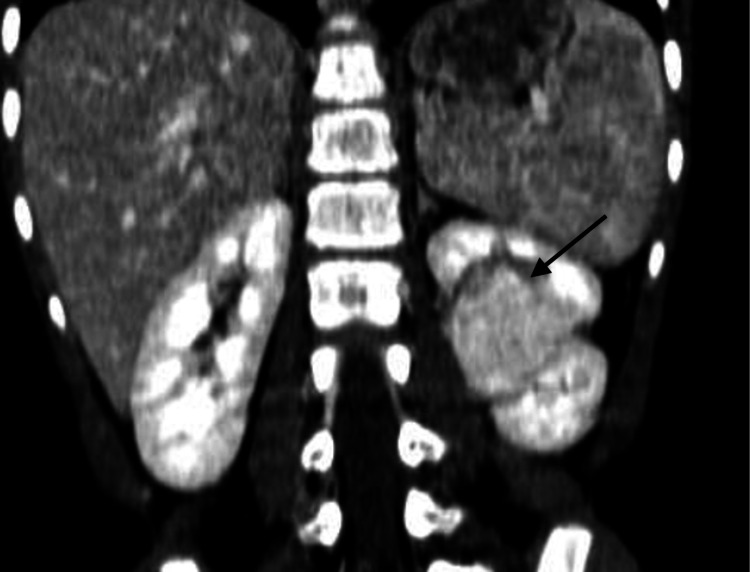
Coronal CT image in the nephrographic phase showing homogeneous enhancement of the lesion

To further characterize the lesion, contrast-enhanced CT was performed. It demonstrated a homogeneous soft-tissue mass in the interpolar region of the left kidney, protruding into the renal sinus. The lesion was isoattenuating to the renal cortex across all contrast phases and preserved the normal renal contour without signs of necrosis or capsular distortion (Figures 2-3).

Based on these imaging features, a diagnosis of an HCB was made. Given the benign imaging appearance and absence of clinical symptoms, the patient was managed conservatively with scheduled ultrasound follow-up. At six months, the lesion remained stable in size and echotexture. The patient remained asymptomatic and was discharged with recommendations for continued non-invasive follow-up.

## Discussion

The HCB is a well-recognized renal pseudotumor that can closely mimic neoplastic lesions, particularly in pediatric imaging, where anatomical variants are more prevalent and the kidneys are relatively smaller in size [3]. It represents a deep inward extension of normal cortical tissue, most commonly located between the upper and middle calyces [4].

On imaging, the HCB typically appears isoechoic to the renal cortex on ultrasound and isoattenuating on computed tomography (CT), maintaining normal corticomedullary continuity. However, focal hyperechoic areas may also be observed in some cases, which are suggested to represent interlobular vessels coursing through the hypertrophied cortical tissue. Key features that help differentiate it from true renal tumors include a smooth interface with the renal sinus, preservation of the overall renal contour, and homogeneous contrast enhancement [5,6].

In pediatric patients, the differential diagnosis includes Wilms tumor, mesoblastic nephroma, lymphoma, and, less commonly, focal pyelonephritis. Wilms tumor often demonstrates heterogeneous enhancement, collecting system distortion, or capsular bulging. Lymphoma generally appears as multiple, poorly enhancing lesions with minimal mass effect [7].

Magnetic resonance imaging (MRI) is particularly helpful in pediatric cases due to its excellent soft tissue contrast and lack of ionizing radiation. HCB on MRI typically shows identical signal intensity to the renal cortex on both T1- and T2-weighted images and demonstrates similar enhancement after contrast administration. Contrast-enhanced ultrasound (CEUS) offers a radiation-free alternative, with homogeneous enhancement patterns comparable to surrounding renal parenchyma, making it a valuable tool for evaluating suspected pseudotumors in children [8].

In our case, neither MRI nor CEUS was performed due to the lack of availability of these modalities in our department. Nevertheless, their diagnostic value is well recognized in the literature.

Although these imaging features have been described in earlier studies [9], recent case-based reports continue to highlight the importance of multimodality imaging in the accurate identification of an HCB. For example, Mullineux described a pediatric case in which CT and MRI findings confirmed a prominent column of Bertin, thereby avoiding unnecessary treatment [10].

In our case, the lesion’s continuity with the renal cortex, its homogeneous and symmetric enhancement, and its stability over time were reassuring features consistent with a benign pseudotumor. On ultrasound, the lesion appeared as a well-defined, isoechoic cortical projection extending into the renal sinus, with no distortion of the renal contour or evidence of mass effect. Its echogenicity was identical to that of the surrounding renal parenchyma, and no signs of calcification, necrosis, or capsule formation were observed. These findings are consistent with an HCB. This variant is best recognized by its isoechoic appearance, central location within the renal sinus, and preservation of normal renal architecture.

Recognition of these imaging characteristics is essential to avoid misdiagnosis and unnecessary interventions, particularly in pediatric patients. In such cases, once the diagnosis is confirmed with cross-sectional imaging, follow-up can typically be performed using ultrasound alone, thereby avoiding repeated radiation exposure.

## Conclusions

The HCB is a common and benign anatomical variant of the kidney that can closely mimic neoplastic lesions, particularly on ultrasound. Recognizing its characteristic imaging features - such as cortical continuity, homogeneous contrast enhancement, and absence of mass effect - is essential to avoid unnecessary diagnostic procedures or surgical interventions. In asymptomatic patients with typical imaging findings, a multimodal imaging approach combined with conservative follow-up represents an appropriate and safe management strategy.
